# 24 GHz Flexible Antenna for Doppler Radar-Based Human Vital Signs Monitoring

**DOI:** 10.3390/s21113737

**Published:** 2021-05-27

**Authors:** Nitin Kathuria, Boon-Chong Seet

**Affiliations:** Department of Electrical and Electronic Engineering, Auckland University of Technology, Auckland 1010, New Zealand; nkathuria.niet@gmail.com

**Keywords:** flexible antenna, Doppler radar, human vital signs, noncontact monitoring, bio-radar, liquid crystal polymer, bending analysis

## Abstract

Noncontact monitoring of human vital signs has been an emerging research topic in recent years. A key approach to this monitoring is the use of the Doppler radar concept which enables real-time vital signs detection, resulting in a new class of radar system known as bio-radar. The antennas are a key component of any bio-radar module and their designs should meet the common requirements of bio-radar applications such as high radiation directivity and mechanical flexibility. This paper presents the design of a four-element antenna array on a flexible liquid crystal polymer (LCP) substrate of 100 μm thickness and ε_r_ of 3.35. The designed antenna array can be used with a 24 GHz bio-radar for vital signs monitoring in a non-contact manner. It features a relatively compact size of 36.5 × 53 mm^2^ and measured gain of 5.81 dBi. The two vital signs: breathing rate (BR) and heart rate (HR) of two human subjects are detected with relatively good accuracy using the fabricated antenna array and radio frequency (RF) output power of −3 dBm from a distance of approximately 60 cm. The effect of bending on the antenna performance is also analyzed.

## 1. Introduction

In today’s hectic pace of life, there is an increasing risk of lifestyle disorders such as cardiovascular diseases, hypertension, and diabetes. Hence, the measurement of physiological signals for the timely detection of such disorders has important applications in the healthcare sector. The vital signs of interest in these applications often include heart rate (HR) and breathing rate (BR). Most conventional devices for measuring vital signs are in contact with the subject, which requires the sensing probes to be physically attached to the patient and instrument, thereby limiting the movements of the patient. Hence, there is a need to research a better solution with similar accuracy to conventional devices. While wearable sensors offer greater freedom of movements to patients, they still need to be in contact with the subject and may not be always comfortable to wear.

The above reasons motivated recent investigations into contactless-based sensing, and radar-based sensing at microwave frequencies [1] appears to be a promising alternative to conventional implantable or wearable sensors for vital signs monitoring. This leads to the concept of bio-radar [2], which promises to revolutionize the healthcare industry. In addition to evaluating the state of health and recovery process of a patient, such radars are also used for sleep-stage estimation and sleep quality analysis through vital signs and sleep movement monitoring [3]. Furthermore, they can be deployed to detect signs of life in post-disaster search and rescue operations [4].

This concept uses Doppler radar to capture the BR and HR information in a contactless way. In a typical radar system, two antennas are used: one for the transmitter and another for the receiver. The transmitting antenna focuses the signal to the chest of the subject and the receiving antenna acquires the reflected signal, whose frequency changes due to micro-movements of the subject’s chest caused by breathing and heartbeat. This is known as the Doppler effect, in which the received frequency increases or decreases as the target moves towards or away from the radar, respectively [5].

One cycle of a heartbeat involves contracting and pumping blood through the circulation system between the heart and the rest of the body [6]. On the other hand, the respiration is a two-step quasi periodic activity which involves inhalation and exhalation [7]. The HR and BR information embedded in the received radar signal can be extracted using an appropriate digital signal processing algorithm.

The antennas are a key component of any bio-radar module as transmission and reception of the signal with minimum losses is the key to a successful operation. They can be composed of a single element or an array of multiple elements, can be designed for different frequencies, and use different types of substrates. However, their designs should meet the application requirements of the bio-radar, which include high radiation directivity, physical compactness, and mechanical flexibility, among others.

To the best of our knowledge, no flexible bio-radar system has been proposed in the existing literature. The flexibility of the antennas, which act as sensors in the bio-radar system, can enable them to be mounted on even non-planar surfaces such as curved walls and edges that may exist in the surroundings of the patient due to their inherent conformity. In turn, this can enable such bio-radar systems to achieve a measurement accuracy closer to that of conventional devices.

In the initial phase of our work presented in [8], we designed and simulated a flexible single antenna element (also known as a unit cell) for a 24 GHz bio-radar system. This was followed by the design and simulation of a two- and four-element array using the same unit cell to achieve better gain performance (higher directivity and efficiency) for the bio-radar application. This paper presents our further work where the designed antennas are fabricated, measured, and utilized in a test setup to evaluate the accuracy of the measured BR and HR acquired by the antennas from real human subjects.

The rest of this paper is organized as follows. Section 2 reviews the related works on antennas for radar applications. Section 3 provides the preliminaries on bio-radar theory and vital signs measurement. Section 4 presents the proposed antenna designs, including a unit cell and its two- and four-element arrays. Section 5 discusses their simulated and measured results. Finally, Section 6 concludes the paper.

## 2. Related Works

This section discusses related works on antenna designs for radar applications. The discussions are categorized by the radar’s antenna requirements, namely operating frequency, radiation directivity, physical compactness, and mechanical flexibility requirements.

### 2.1. Operating Frequency

Radar modules can be designed for low, intermediate, and high frequency. In [9], a 2 × 2 patch antenna array was implemented on low-temperature co-fired ceramic (LTCC) substrate and was integrated with a 60 GHz Doppler radar system. In [10], a 24 GHz millimeter wave antenna was designed to monitor the vital signs of patients who are immovable and in mental stress. Another design operating at 77 GHz in [11] is a linear array with ten small patch antennas connected with quarter wave impedance transformers to reduce sidelobe levels. A 915 MHz fractal antenna used with a low noise amplifier was proposed for a continuous wave (CW) Doppler radar to monitor heart rate and breathing rate [12]. However, the use of low frequency limits the achievable antenna compactness and detection sensitivity when operating in a near-field condition. On the other hand, the use of high frequencies can make the bio-radar sensitive to subtle movements of the subject, such as chest wall micromovements due to heartbeat.

### 2.2. Radiation Directivity

The higher the antenna directivity, the higher the received signal-to-noise ratio and the more accurate the measurement by the radar signals. In [13], the authors evaluated different 2.4 GHz antennas for vital signs monitoring, including patch, helical, and Yagi-Uda antennas. The patch antenna exhibits a half-power beamwidth of 92° and a promising performance for vital signs detection. On the other hand, the helical antenna operating in axial mode at 2.4 GHz is an 8-turn design with on-reflector impedance matching to maintain the size. Compared to a conventional 2.4 GHz patch antenna, the helical design can offer a higher gain. However, the planar nature of the patch is attractive for achieving a low profile and ease of fabrication using printed circuit board (PCB) technology. To improve directivity, multi-element array and higher operating frequency can be used. For a given antenna size, a higher frequency narrows the beamwidth due to a larger size-to-wavelength ratio, resulting in higher directivity. The narrower beamwidth allows the antenna aperture to focus towards the human subject for transmission and reception, thus avoiding interference from environmental clutter and noise.

### 2.3. Physical Compactness

The compactness of the antenna is important for some radar applications such as in portable radar deployment or when an antenna array is needed to achieve the desired performance characteristics or multiband operation. Designing an antenna to operate at a high frequency can reduce not only antenna size, but also spacing between collocated antennas (e.g., transmit and receive antennas) of the radar system that must be at least a half wavelength to avoid mutual coupling. A compact radar system can also be achieved by using only one antenna for both transmit and receive [14]. However, in such systems, circulators are often used to separate the transmitted and received signals, which are not compact in size and expensive. An alternative is the use of couplers [15], but they divide signal power and add to insertion loss, rendering the radar system more prone to noise and interference. In [16], a comparative analysis is performed on a bio-radar using single- and two-antenna designs. Results showed that while the single-antenna system performed well, the detection range was somewhat limited. On the other hand, the performance of two-antenna system was good without the drawback of a limited range. Hence, for applications where a long detection range and compactness are both essential, the key challenge is to design a two-antenna radar system with a relatively small footprint.

### 2.4. Mechanical Flexibility

Flexibility is another upcoming property of the flexible circuits for next-generation consumer electronics. The materials used may include soft plastics, textiles, or even paper to make conformal antennas that can be easily integrated onto non-planar surfaces [17] such as spherical, cylindrical, and other complex shaped surfaces. In [18], the authors designed a 2.45 GHz wearable antenna using flexible material to measure human vital parameters. The materials included fabrics, Teflon, rubber, and paper. In [19], the bending effects on a flexible ultra-wide band antenna on liquid crystal polymer (LCP) substrate were studied. It demonstrated the antenna’s ability to maintain its radiation pattern and gain performance under various bent conditions. However, these are wearable antennas that operate in near-field conditions. To the best of our knowledge, the flexibility feature has not been explored by bio-radar systems that operate in the far-field for vital sign monitoring. Such flexibility is also desired for non-wearable antennas so that they can be mounted on any surfaces.

The findings from the above review motivated us to propose a bio-radar antenna with the following features: (i) high operating frequency (24 GHz); (ii) high directivity using multi-element patch array; (iii) compact low-profile two-antenna design; and (iv) flexibility to allow mounting on non-planar surfaces such as curved walls and edges.

## 3. Preliminaries

### 3.1. Bio-Radar Theory

In this bio-radar concept, electromagnetic waves are used to monitor the vital signs. It works on the principle of the Doppler effect, in which a transmitter sends a signal and a receiver captures a signal with a change of phase and polarization upon reflection from a subject’s body [2]. Moreover, there is a change in the frequency of the signal due to micromovements of the subject’s chest caused by breathing and heartbeat. The distance traveled by the radar signal from transmission (TX) to reception (RX) can be expressed in terms of the number of wavelengths $N_{\lambda }$, which can be calculated as:

(1) $$N_{\lambda }=\frac{2R}{\lambda }$$

where *R* is the one-way distance between bio-radar and target subject, and λ is the signal wavelength. Under the Doppler effect, if both the radar and the target are fixed, then the received frequency does not change, as *N_λ_* is constant. However, if the target is moving, either in the direction of or away from radar, then the received frequency increases, and decreases, respectively [5]. This frequency shift is due to the change in wavelength, which is in turn due to the change in distance, as with each wavelength there is a phase change of 2*π*. Hence, this phase change α is dependent on distance as:

(2) $$\alpha =2\pi N_{\lambda }$$

In literature, radars can be generally classified into continuous-wave or impulse radar. The latter is also known as ultra-wide band (UWB) radar. Continuous-wave radar can be further classified into unmodulated continuous-wave (CW) or frequency-modulated continuous-wave (FMCW) radar. Continuous-wave and UWB are also two common modes of operation of bio-radar for vital signs measurements.

A narrow band single tone signal is transmitted and received in CW radar. It can monitor and evaluate the velocity of a subject’s movements. This enables it to differentiate between a moving and a stationary target within its range [20]. The signal processing system is simple because of narrow band signals. However, there may be a signal interference from transmitter side to receiver side due to some signal leakage. Moreover, there may be reflections from other objects in the vicinity, which can interfere with the received signal, leading to a low signal-to-noise ratio.

The FMCW radar can monitor distance and velocity between the radar and the target, which is not the case with the CW radar. Here, a triangular modulation is used to vary the signal frequency linearly with respect to time, and another triangular modulation is used with the received signal. The delay time T between the TX and RX signals is given by:

(3) $$T=\frac{2R}{c}$$

where *c* is the speed of light. The bandwidth of modulated signal determines the distance measurement accuracy, and the rate of modulation determines the maximum detectable distance without ambiguity. The frequency difference between TX and RX signals in FMCW is due to the target movement. Hence, through this distance, the vital signs can be extracted.

On the other hand, the UWB radar generates very short pulses to transmit information over a very wide spectrum. For a signal with a bandwidth higher than 25% of its center frequency, it is considered to be a UWB signal. These transmitted short pulses are reflected back by the target, and the smallest target movement distance ΔR that can be detected, i.e., range resolution of the radar, can be calculated as [21]:

(4) $$\Delta R=\frac{c}{2BW}=\frac{c\tau }{2}$$

where *BW* is the bandwidth of radar pulse, and τ is the pulse width. The distance-to-target $D_{t}$ is given by (5) where Δt is the time delay between the TX and RX signals:

(5) $$D_{t}=\frac{c\Delta t}{2}$$

### 3.2. Vital Signs Measurement

The measurement of vital signs such as heart rate (HR) and breathing rate (BR) are based on micro-movements of the chest that can be monitored using radar operating at microwave frequencies [22]. The signal is transmitted through an antenna onto the human chest, which is then reflected with the information of chest displacement caused by the heartbeat and lung respiration (breathing).

The heartbeat is produced due to the pumping of blood in the whole body. The blood carries oxygen and energy to the cells. The cardiac cycle has a sequence of events through pulmonary and systemic circuits. There are two main episodes of the process: diastole and systole. In this process, the heart is the muscle that does the pumping of the blood and the blood vessels act as the transporters [23]. The pumping causes periodic contraction and enlargement of the heart, leading to micro-displacements on the chest walls.

Breathing is a two-step quasi periodic activity that involves inhalation of oxygen and exhalation of carbon dioxide. Due to this exchange of air in the lungs, there is a contraction of muscles caused by the pressure difference between the thorax and the external atmosphere. The movement of this air to and from the lungs causes displacement in the chest region including the ribcage, the abdomen, and the thorax, as well as on the outer surface of the skin. This displacement is measured by the Doppler effect through a bio-radar. The average chest displacement due to heartbeat and breathing is 0.2–0.5 mm and 4–12 mm, respectively [24].

**Figure 1:**
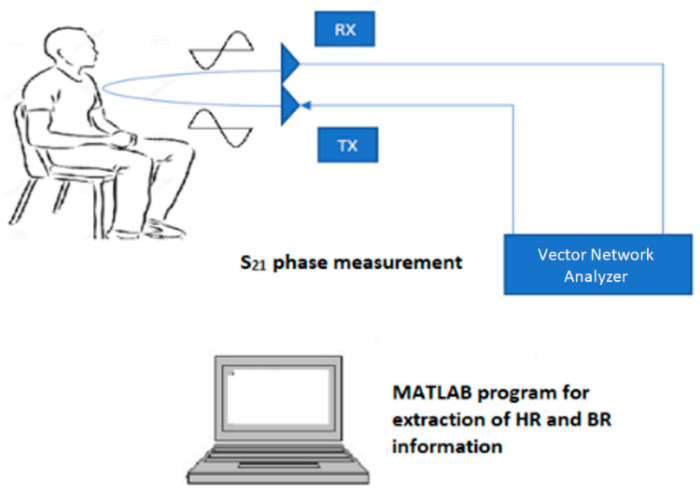
Setup for HR and BR measurements.

Figure 1 shows a typical setup for HR and BR measurements, which is also used in this paper. Two antennas, one for TX and one for RX, are positioned with their radiating element facing the chest area of a subject sitting at an appropriate distance away. The TX and RX antennas are connected to Port 1 and Port 2 of the vector network analyzer (VNA), respectively. The VNA is set up to measure the S_21_ parameter, which represents the signal reflected from the subject detected by the RX antenna relative to the incident signal transmitted by the TX antenna. For HR and BR measurements, knowing the phase of S_21_ is the most critical, as the phase variation provides information about the chest displacement. This phase change Δθ(t) can be found by [25].

(6) $$\Delta \theta \left(t\right)=\frac{4\pi }{\lambda }\Delta x\left(t\right)$$

where Δ*x*(*t*) is the chest displacement. The obtained S_21_ data is then processed in three steps that can be implemented in a Matlab program to extract the HR and BR information. The three processing steps are: (i) filtering to remove any unnecessary information; (ii) fast Fourier transform (FFT) to convert the time-domain phase signal into the frequency domain; and (iii) peak detection in the frequency spectrum to determine the HR and BR information. The following outlines in more detail each of these processing steps:

- *Filtering*: Digital bandpass filtering is performed on received raw S_21_ phase signal with cutoff frequencies appropriately defined to pass the range of frequencies corresponding to the expected range of HR and BR.
- *Fast Fourier transform*: FFT decomposes the time-varying S_21_ phase signal into its constituting frequency components. A magnitude plot of the frequency components is then obtained, and peak detection is performed to extract the HR and BR values.
- *Peak detection*: In the FFT spectrum, the frequency of the highest peak between 0–0.34 Hz identifies the breathing rate (corresponding to 0–20 breaths per minute), while the frequency of the highest peak between 1–2 Hz identifies the heart rate (corresponding to 60–120 beats per minute). Prior to the peak detection, the frequency range of 0–2 Hz is commonly converted into a vital signal range of 0–120 beats/breaths per minute.

## 4. Proposed Antenna Designs

For effective detection of human HR and BR using a bio-radar system, it is important to utilize antennas that operate at high frequencies, e.g., in the super high frequency range of 3–30 GHz; exhibit high gain, i.e., high directivity and electrical efficiency; are compact to deploy a two-antenna system with good detection range; and flexible to allow conformal mounting on surfaces of any geometry such as curved walls and edges.

Hence, three flexible patch antennas were designed to operate at 24 GHz: (i) single-element unit cell; (ii) two-element array; and (iii) four-element array. The initial square patch antenna dimensions were calculated, and then simulations were conducted to optimize the design in terms of size and performance, resulting in an ellipse-shaped slotted patch unit cell. Two- and four-element arrays were then designed based on this optimized unit cell. The aim of increasing the number of elements was to improve the gain of the antenna.

LCP was the flexible substrate material chosen for our antennas. Compared to other flexible materials such as fabrics, Teflon, rubber, and paper, LCP exhibits better dielectric stability and lower transmission line losses at high frequencies. The operational temperature of LCP can reach 190 °C, which enables multiple and standard re-flow and soldering operations. LCP also exhibits low moisture absorption and chemical stability [26].

### 4.1. Single-Element Unit Cell

**Figure 2:**
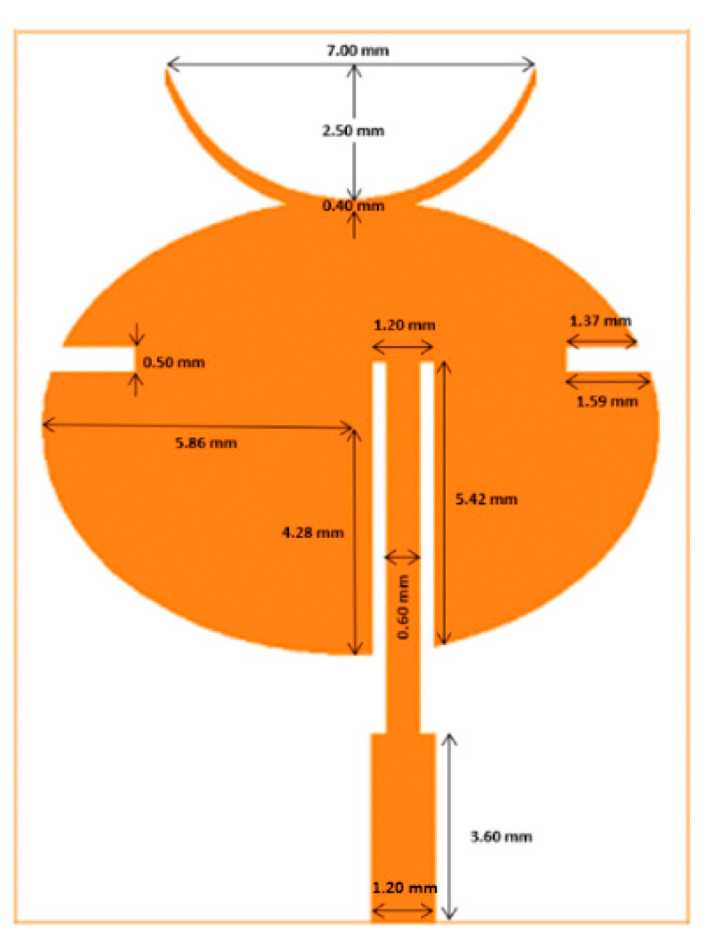
Single-element unit cell design.

The designed unit cell consisted of a monopole patch, feed element, and ground plane with dimensions as shown in Figure 2. The ellipse-shaped radiating patch had a semi-major axis and semi-minor axis length of 5.86 mm and 4.28 mm, respectively. The elliptical shape offers more parametric degrees of freedom in designing the antenna to the desired performance compared to rectangular- and circular-shaped patches. For the purpose of frequency tuning and to achieve polarization symmetry, two slots measuring 0.5 × 1.59 × 1.37 mm were cut on both sides of the horizontal ellipse. In addition, a top horn structure was introduced to enhance the directivity of the unit cell. The copper thickness of the patch and ground plane was 18 μm as specified in the datasheet of the LCP substrate.

### 4.2. Two-Element Array

**Figure 3:**
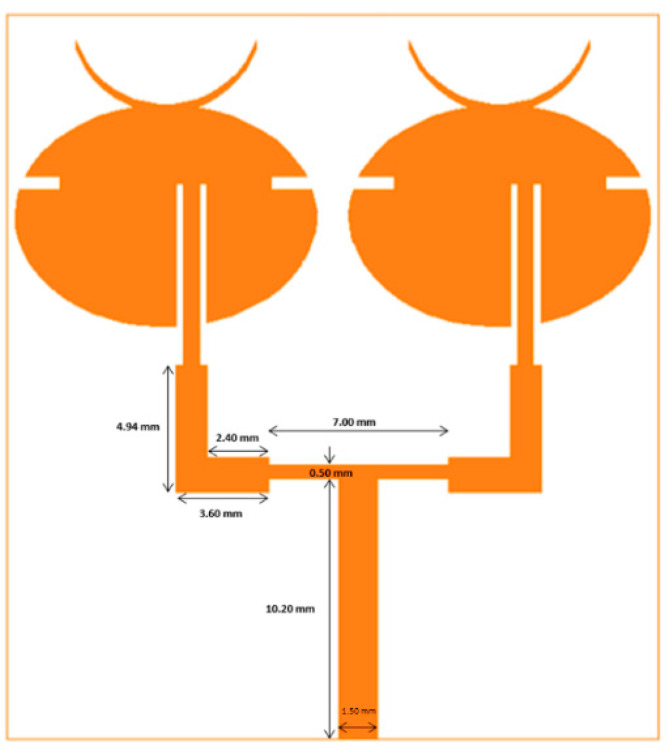
Two-element array design.

To further improve the gain of the unit cell, arrays using multiple unit cells were designed. Figure 3 shows the designed two-element array. The closest separation between the two elements was 1.6 mm. The feed line was a 50 Ω input, which was split into two 100 Ω lines to power each individual patch. Here, the T-shaped power divider was used along with a quarter wave transformer for impedance matching.

### 4.3. Four-Element Array

**Figure 4:**
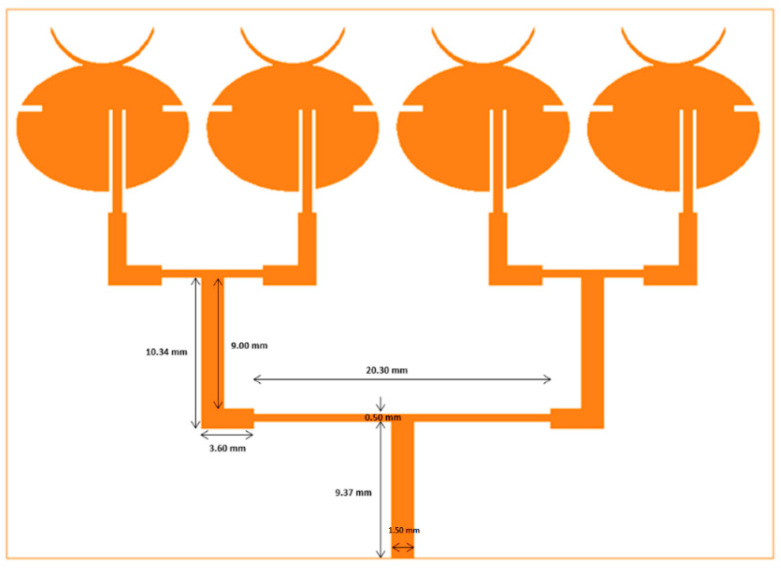
Four-element array design.

**Figure 5:**
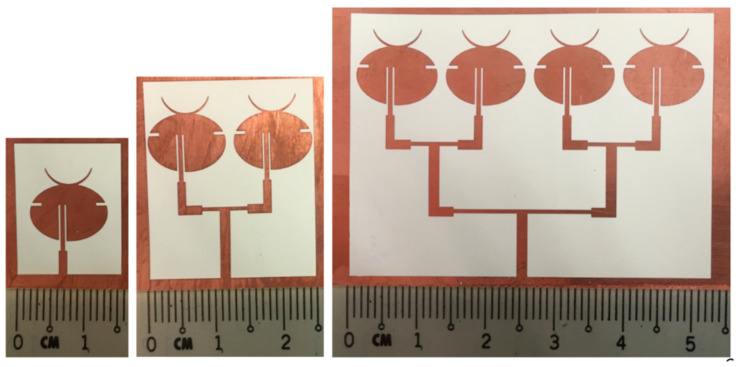
Fabricated antenna prototypes.

Similarly, a four-element array was designed as shown in Figure 4, where inter-element separation was 1.6 mm. Figure 5 shows the prototypes of all three antenna designs fabricated on a copper-clad LCP substrate of thickness 0.1 mm using photoetching. The performance of both simulated and fabricated antennas is analyzed in the next section.

## 5. Results and Discussion

### 5.1. Simulated Antenna Performance

The antennas were designed and simulated in Ansys HFSS where a wave port was used for excitation. Each of the antennas, the single-element unit cell, two-, and four-element array, were evaluated in terms of their return loss, radiation pattern, and gain performances.

**Figure 6:**
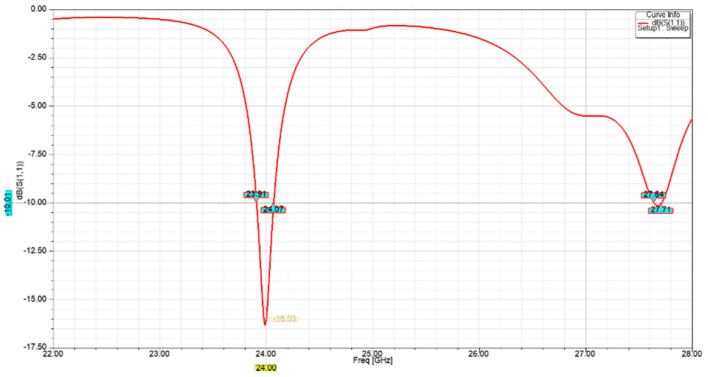
Single-element return loss performance.

The return loss |S_11_| is a measure of the impedance mismatch between the feed line and antenna. The lower the |S_11_|, the less power is reflected and lost through impedance mismatch, and the more power is delivered to antenna. Figure 6 shows that the |S_11_| of the designed single-element unit cell was lower than −16 dB at the desired operating frequency of 24 GHz.

**Figure 7:**
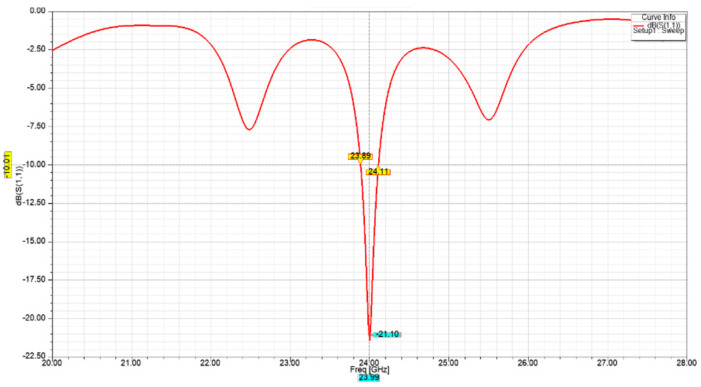
Two-element array return loss performance.

**Figure 8:**
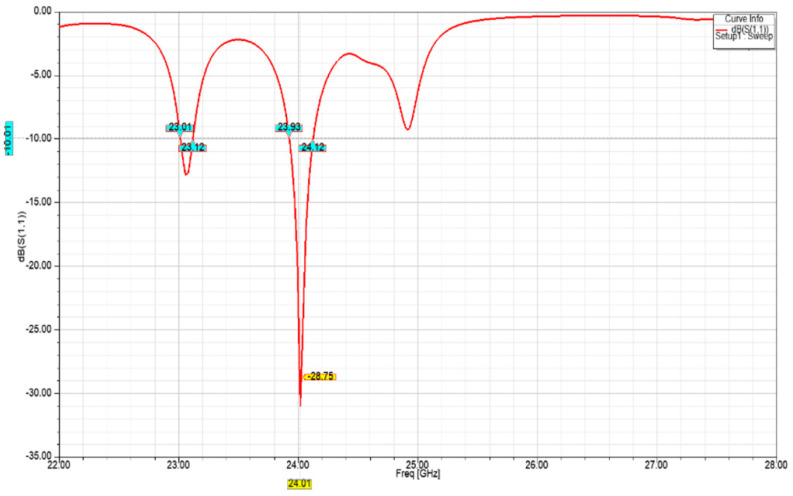
Four-element array return loss performance.

Figure 7 and Figure 8 show an increasingly improved return loss performance of lower than −21 dB and −28 dB for the two-element array, and four-element array, respectively. The latter also exhibits a −10 dB bandwidth of approximately 190 MHz.

**Figure 9:**
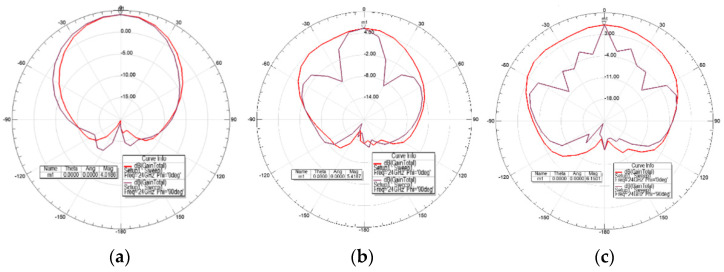
Radiation pattern of (**a**) single-element unit cell; (**b**) two-element array; and (**c**) four-element array. The plot at φ = 0° (red) and φ = 90° (purple) refers to the radiation pattern on E-plane (side-view) and H-plane (top-view), respectively.

**Figure 10:**
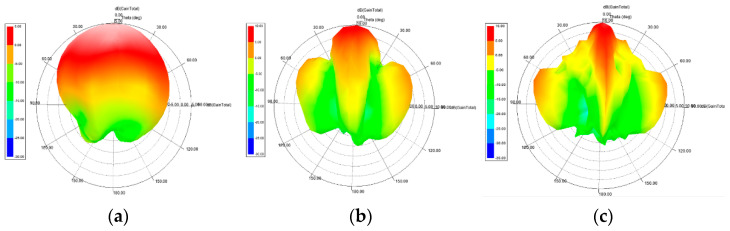
The corresponding 3D polar plot of (**a**) single-element unit cell; (**b**) two-element array; and (**c**) four-element array.

The radiation pattern depicts how the transmitted signal from the antenna radiated in different directions. Figure 9 and Figure 10 show the 2D radiation pattern and the corresponding 3D polar plot, respectively, for each antenna. They show that the maximum radiation intensity was in the direction of θ = 0° for both unit cell and arrays. Moreover, increasing the number of elements had the effect of narrowing the main beam.

**Figure 11:**
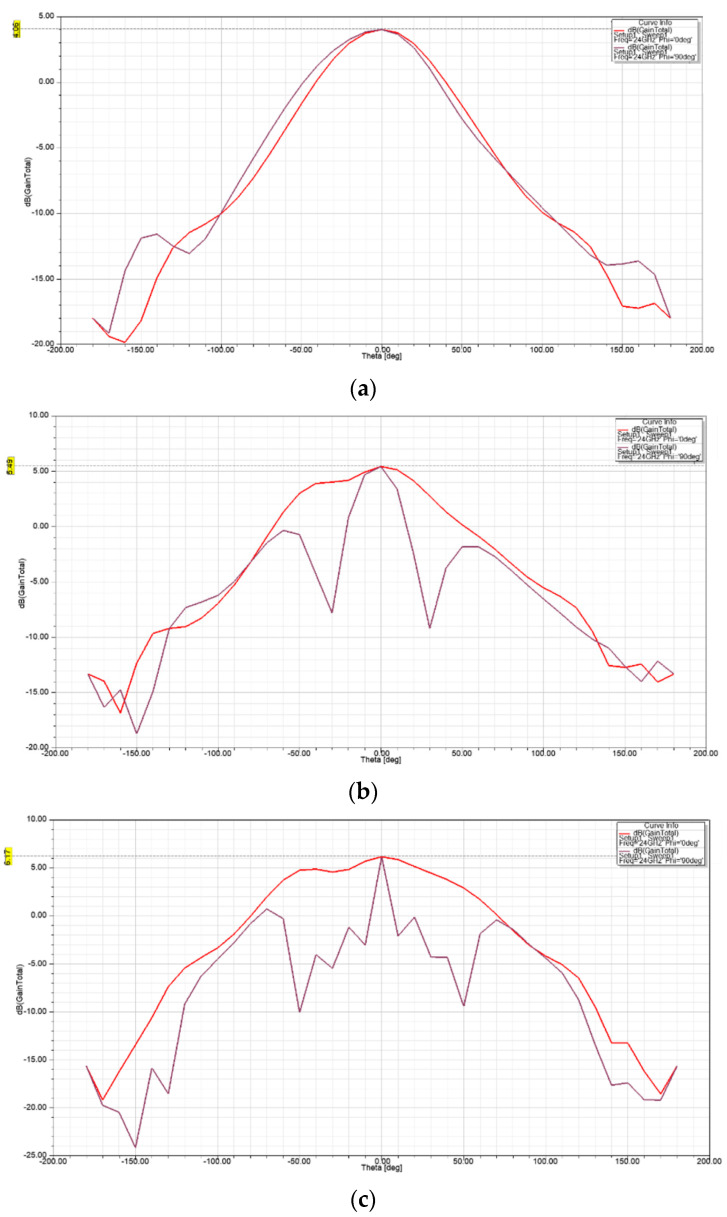
Gain of (**a**) single-element unit cell; (**b**) two-element array; and (**c**) four-element array; as a function of θ at φ= 0° (red) and φ = 90° (purple).

The gain of an antenna is a measure of its directivity or the extent to which its signal propagates in the peak direction of radiation. The gain on E-plane (φ = 0°) and H-plane (φ = 90°) as a function of theta (θ) at 24 GHz for each antenna is shown in Figure 11. The results show a maximum gain of 4.06 dBi, 5.49 dBi, and 6.17 dBi, at θ = 0° on both planes for the single-element unit cell, two-element array, and four-element array, respectively. The achieved gain is attributed to the added horn type structure to the basic elliptical design.

### 5.2. Measured Antenna Performance

**Figure 12:**
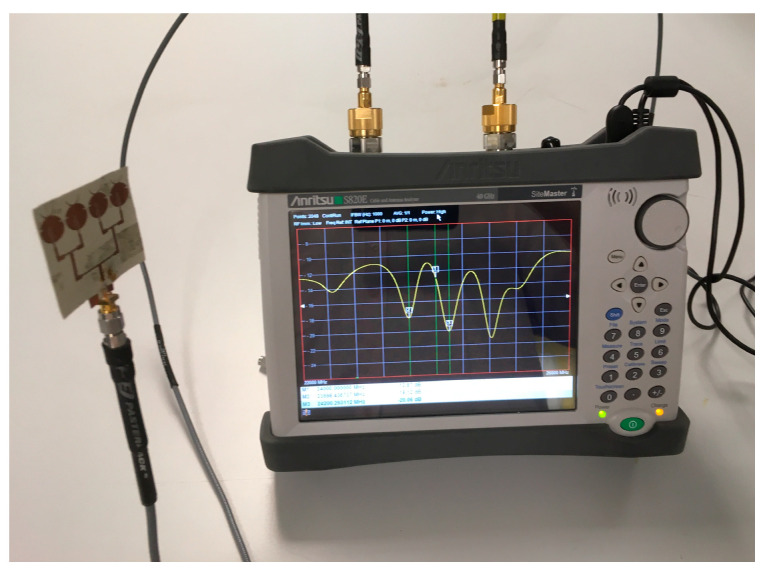
Example return loss measurement of an antenna prototype using the VNA.

This section presents the performances of our fabricated antennas measured using the Anritsu S820E VNA (Anritsu Co., Morgan Hill, CA, USA). Each antenna was interfaced with the coaxial cable of the VNA using a SMA connector with a specified maximum operating frequency of 26.5 GHz. For the gain measurement, a horn antenna with an operating frequency range of 18–26.5 GHz and gain of 15 dBi was used as the reference antenna. Both reference and test antennas were positioned approximately 1 m apart. Figure 12 shows an example of measuring the return loss |S_11_| of an antenna prototype using the VNA.

**Figure 13:**
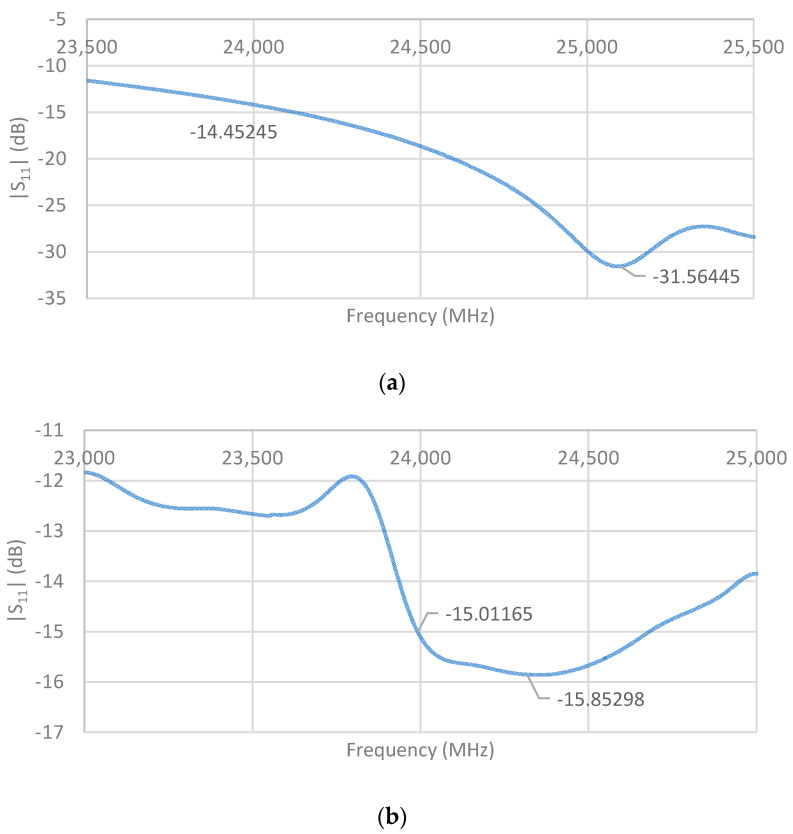

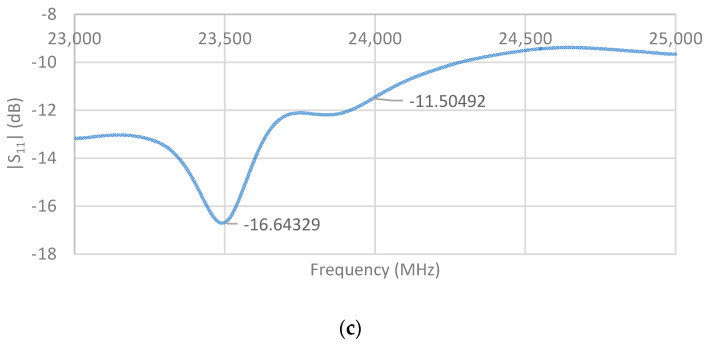
Measured return loss of (**a**) single-element unit cell; (**b**) two-element array; and (**c**) four-element array.

The measured return loss of the single-element unit cell, two-element array, and four-element array are shown in Figure 13. There were apparent differences from the simulated results, which we attribute to fabrication imperfections such as connector positioning and soldering. However, the measured return losses at the desired frequency of 24 GHz were still better than the minimum requirement of −10 dB for all three fabricated antennas.

The gain of each fabricated antenna was measured using the gain transfer method [27]. The measurement steps involved: (i) measuring the S_21_ between reference and test antennas, where S_21_ represents the ratio of received to transmitted power of the antennas measured by the two ports of VNA; (ii) calculating the free-space path loss between reference and test antennas using the Friis formula $P_{loss}^{dB}=20log_{10}\frac{\lambda }{4\pi d}$ where *λ* is the signal wavelength and *d* is the distance between antennas; and (iii) obtaining the gain of the test antenna as: $G_{test}^{dB}=S_{21}^{dB}-P_{loss}^{dB}-G_{ref}^{dB}$.

**Figure 14:**
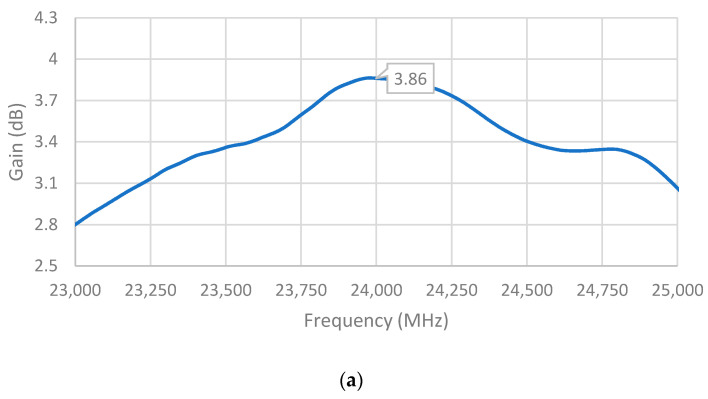

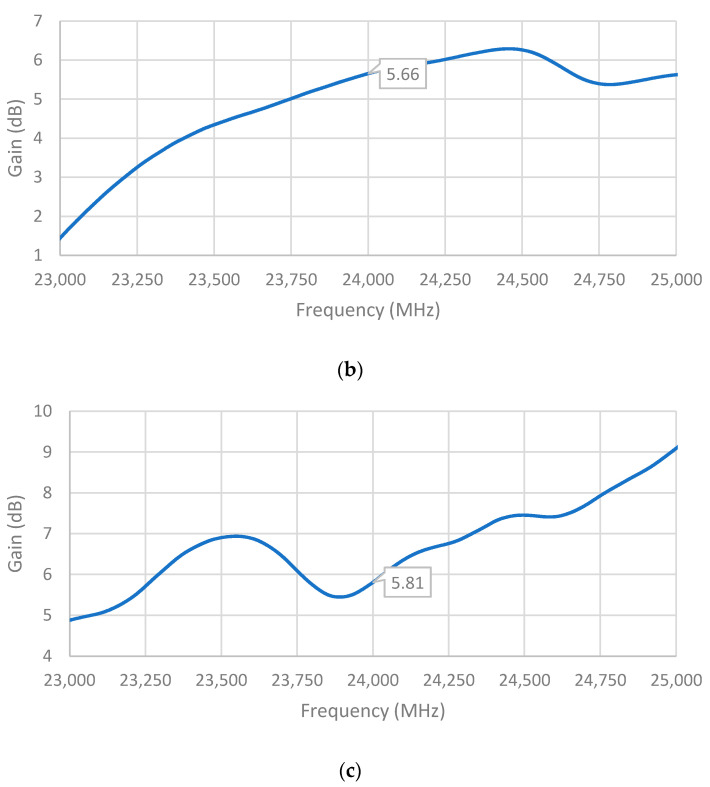
Measured gain of (**a**) single-element unit cell; (**b**) two-element array; and (**c**) four-element array.

The measured gains of the single-element unit cell, two-element array, and four-element array are shown in Figure 14. It can be observed that the maximum gain of the single-element unit cell was achieved at a frequency very close to the desired frequency of 24 GHz, while those of the two-element array and four-element array occurred at higher frequencies. Notwithstanding, the measured gains of all three antennas at 24 GHz were still significant: 3.86 dBi, 5.66 dBi, and 5.81 dBi for the single-element unit cell, two-element array, and four-element array, respectively, which are comparable to the simulated gains.

To further analyze the effect of bending on the antenna performance, the fabricated antennas were positioned on two rolled foams: one with 50 mm, another with 30 mm diameter. Their return loss |S_11_| was measured while bent along their horizontal or vertical axis. The 50 mm foam subjected the two- and four-element arrays to a bending angle of approximately 65° and 105°, respectively, while the 30 mm foam subjected each of the same arrays to a bending angle of approximately 120° and 180°, respectively.

**Figure 15:**
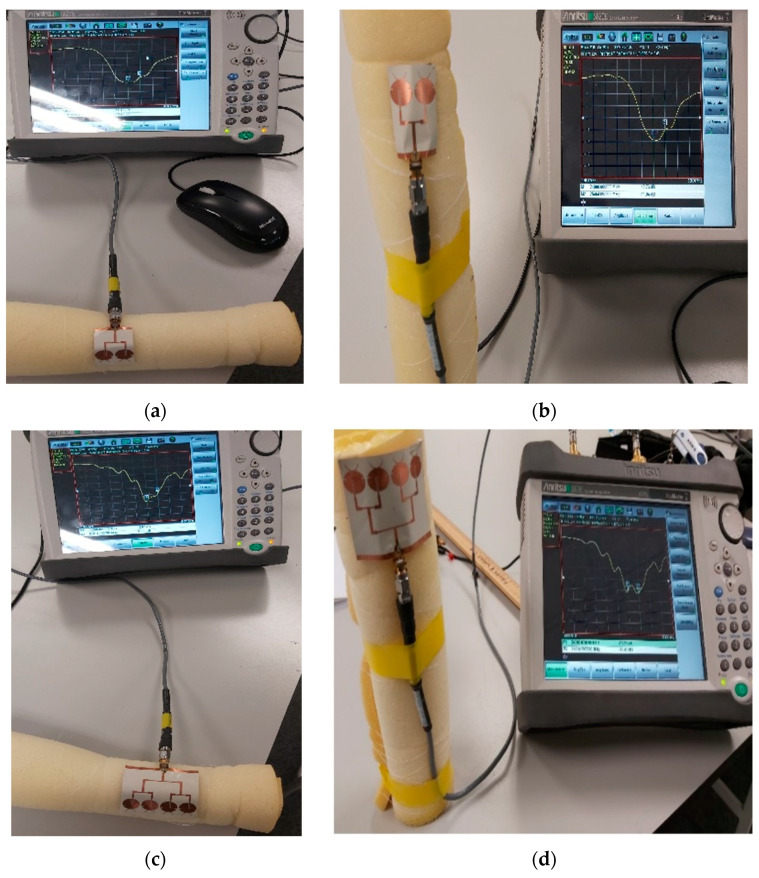
Measurement of two-element (**a**,**b**) and four-element (**c**,**d**) arrays under horizontal (left) and vertical (right) bending on a 50 mm rolled foam.

**Figure 16:**
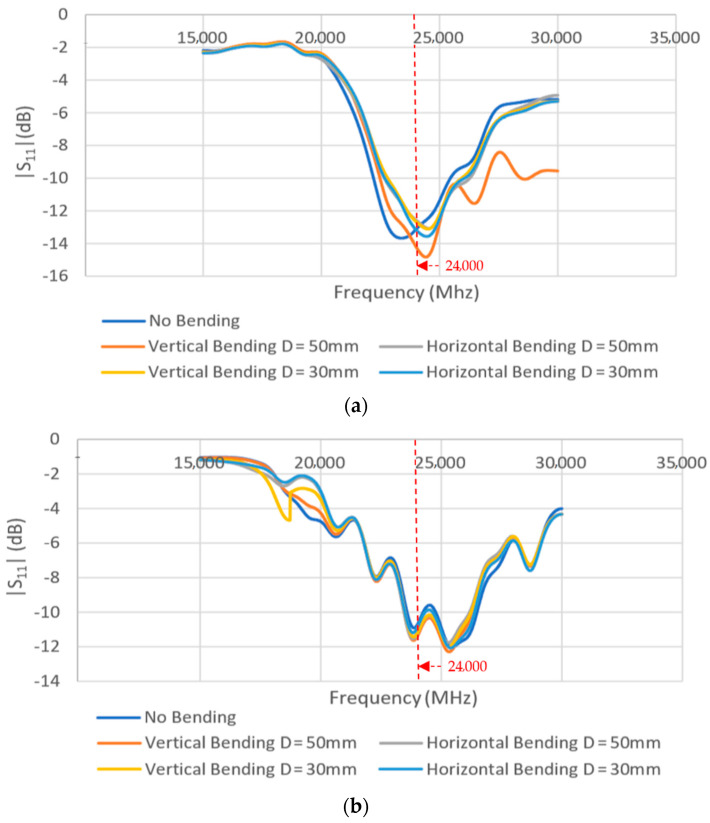
Measured return loss performance for the (**a**) two-element and (**b**) four-element arrays under different bending conditions.

Figure 15 shows the measurement of two- and four-element arrays while bent horizontally (along *x*-axis) and vertically (along *y*-axis) on a 50 mm rolled foam. A comparison of the return loss performance of the arrays under different bending conditions is shown in Figure 16. It was observed that the bending did not significantly alter their performance. The antennas can still achieve reasonable |S_11_| values (<−10 dB) at the desired frequency of 24 GHz.

### 5.3. Measured Heart Rate and Breathing Rate

**Figure 17:**
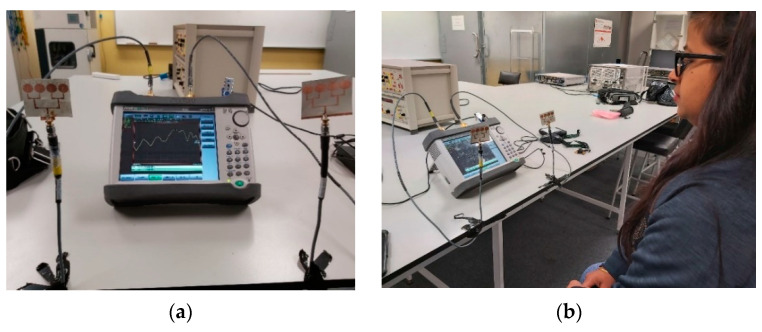
(**a**) Example setup for HR/BR measurement; (**b**) subject sitting in front of the setup.

**Figure 18:**
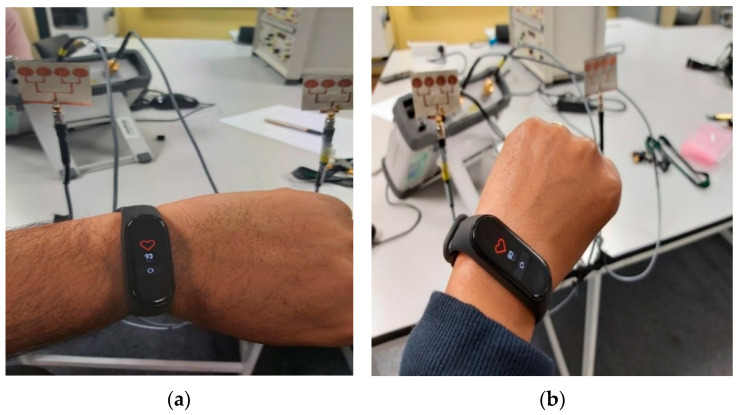
Example measurement of true HR by (**a**) Subject 1 (male) and (**b**) Subject 2 (female); using a wrist-worn HR monitor.

Figure 17 shows an example setup using 2 four-element arrays (one for TX and one for RX) to measure the heart rate (HR) and breathing rate (BR) of a subject sitting approximately 60 cm from the antennas. As mentioned earlier, the setup measured the phase of S_21_, which was then processed by a MATLAB program to extract the HR and BR information. The HR/BR of two subjects (one male, one female) were measured and the effectiveness of both the two- and the four-element arrays were evaluated. For each subject, the estimated HR/BR were averaged over three measurements (each acquires 2048 signal samples over 18 s) and compared with their ground truth. The true HR was measured by a wrist-worn HR monitor (Figure 18), while the true BR was manually monitored by the subjects counting the number of breathing cycles during the measurement.

Table 1 compares the estimated HR/BR with their true values. Subject 1 (male) clearly had a higher HR than Subject 2 (female), which was correctly detected by our radar system. Generally, the estimated HR/BR were comparable with their true values. However, the percentage error in the estimated BR was observed to be higher than that in the estimated HR. We believe that this was not due to a lower BR detection accuracy, but due to the smaller BR values. For example, with a true BR of 15 and estimated BR of 14, the relative error is 6.67%, but with a true HR of 85 and estimated HR of 84, the relative error is only 1.18% (smaller by more than five times). It was also observed that measurements using four-element arrays give generally more accurate estimations than using the two-element array.

**Figure 19:**
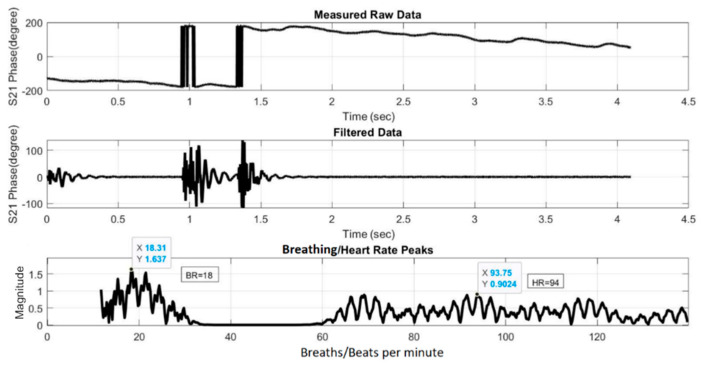
HR/BR measurement of Subject 1 using two-element array (HR: 94, BR: 18).

**Figure 20:**
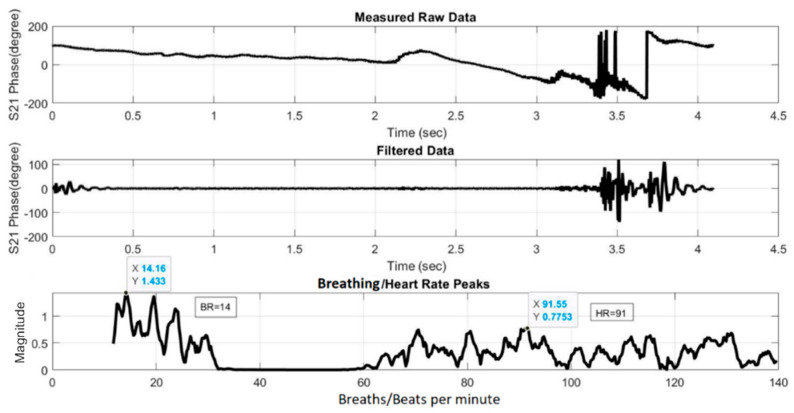
HR/BR measurement of Subject 1 using four-element array (HR: 91, BR: 14).

**Figure 21:**
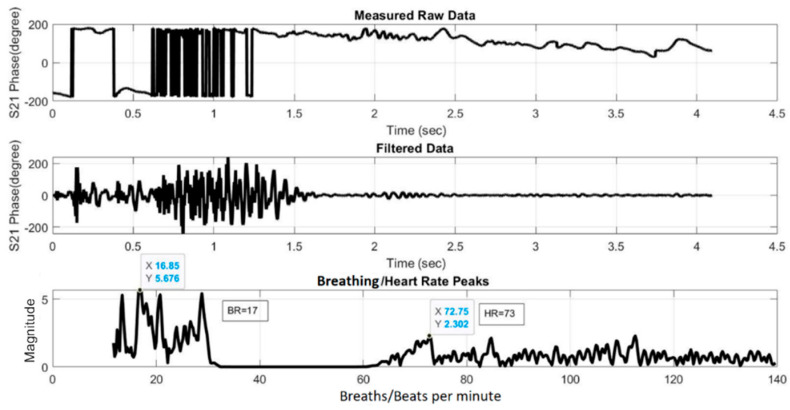
HR/BR measurement of Subject 2 using two-element array (HR: 73, BR: 17).

**Figure 22:**
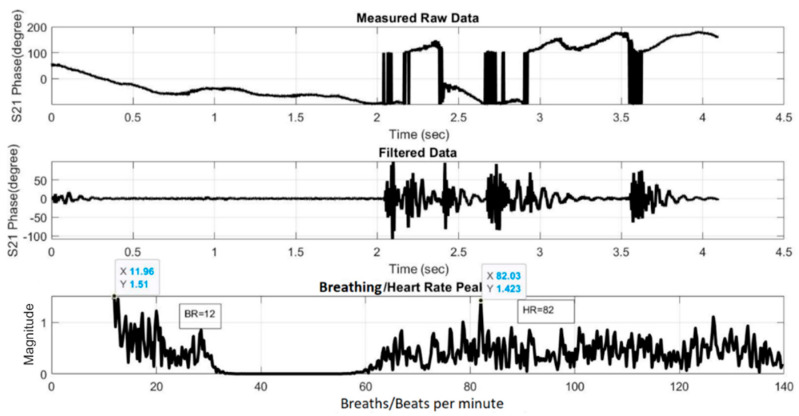
HR/BR measurement of Subject 2 using four-element array (HR: 82, BR: 12).

Figure 19 and Figure 20 show the HR/BR measurements for Subject 1 using the two- and four-element array, respectively. The top figure shows the raw signal of the measured S_21_ phases. The middle figure shows the filtered signal, while the bottom figure shows the transformed signal in frequency domain after FFT. The *x*-axis value of the first highest peak in the *x* = 0–20 range identifies the estimated BR, while that of the highest peak in the *x* = 60–120 range identifies the estimated HR. Figure 21 and Figure 22 show the equivalent measurements for Subject 2.

## 6. Conclusions

This paper firstly presented the design of a single-element unit cell that features an elliptical and horn-shaped patch on flexible LCP substrate for a 24 GHz bio-radar. The design of two- and four-element arrays using the same unit cell were then presented. The four-element array exhibited the best simulated performance in terms of return loss and gain. It was also flexible and thus could conform to the shape of any surface on which it was mounted.

The designed antennas were fabricated, measured, and tested with human subjects to detect their heart and breathing rates. The return losses |S_11_| of all three fabricated antennas were found to be better than the minimum recommended threshold of −10 dB at the desired operating frequency of 24 GHz. The heart and breathing rates of the two human subjects estimated using the S_21_ phase information acquired by the fabricated two- and four-element arrays were found to be reasonably accurate.

Presently, the detection requires the antennas to be positioned in front of a stationary subject with an unobstructed view of the chest area. To be feasible for a broader range of applications, the bio-radar should still perform well when subjects are arbitrarily oriented relative to the antennas or when they are in arbitrary poses and movements. Hence, some potential directions for future work may include: (i) designing a bio-radar antenna array with a steerable beam pattern; (ii) integrating posture and motion with vital signs detection; and (iii) cooperative vital signs monitoring between bio-radars in a distributed environment, akin to the concept of a multi-static radar network.

## Figures and Tables

**Table 1:** Estimated and true HR and BR of two human subjects.

Subject	Antenna Elements	Heart Rate (HR) (Heartbeat per Minute)			Breathing Rate (BR) (Breath per Minute)		
		Estimated	True	Error (%)	Estimated	True	Error (%)
Subject 1 (male)	2	97.33	92	5.79	13	15	13.33
	4	98	93	5.38	14.33	15	4.47
Subject 2 (female)	2	79	76	3.95	16.33	18	9.28
	4	81.67	83	1.60	13.67	17	19.59

## Data Availability

The data that support the findings of this study are available from the corresponding author upon reasonable request.
